# Development of Novel siRNA Therapeutics: A Review with a Focus on Inclisiran for the Treatment of Hypercholesterolemia

**DOI:** 10.3390/ijms24044019

**Published:** 2023-02-16

**Authors:** Oluwakemi Ebenezer, Pietro Comoglio, Gane Ka-Shu Wong, Jack A. Tuszynski

**Affiliations:** 1 Department of Chemistry, Faculty of Natural Science, Mangosuthu University of Technology, Umlazi 4031, South Africa; 2 Department of Mechanical and Aerospace Engineering (DIMEAS), Politecnico di Torino, 10129 Turin, Italy; 3 Department of Biological Sciences, University of Alberta, Edmonton, AB T6G 2E9, Canada; 4 Li Ka Shing Institute of Virology, University of Alberta, Edmonton, AB T6G 2E1, Canada; 5 Department of Physics, University of Alberta, Edmonton, AB T6G 2E1, Canada

**Keywords:** inclisiran, GalNAc-conjugated, siRNA, low-density lipoprotein cholesterol

## Abstract

Over the past two decades, it was discovered that introducing synthetic small interfering RNAs (siRNAs) into the cytoplasm facilitates effective gene-targeted silencing. This compromises gene expression and regulation by repressing transcription or stimulating sequence-specific RNA degradation. Substantial investments in developing RNA therapeutics for disease prevention and treatment have been made. We discuss the application to proprotein convertase subtilisin/kexin type 9 (PCSK9), which binds to and degrades the low-density lipoprotein cholesterol (LDL-C) receptor, interrupting the process of LDL-C uptake into hepatocytes. PCSK9 loss-of-function modifications show significant clinical importance by causing dominant hypocholesterolemia and lessening the risk of cardiovascular disease (CVD). Monoclonal antibodies and small interfering RNA (siRNA) drugs targeting PCSK9 are a significant new option for managing lipid disorders and improving CVD outcomes. In general, monoclonal antibodies are restricted to binding with cell surface receptors or circulating proteins. Similarly, overcoming the intracellular and extracellular defenses that prevent exogenous RNA from entering cells must be achieved for the clinical application of siRNAs. *N*-acetylgalactosamine (GalNAc) conjugates are a simple solution to the siRNA delivery problem that is especially suitable for treating a broad spectrum of diseases involving liver-expressed genes. Inclisiran is a GalNAc-conjugated siRNA molecule that inhibits the translation of PCSK9. The administration is only required every 3 to 6 months, which is a significant improvement over monoclonal antibodies for PCSK9. This review provides an overview of siRNA therapeutics with a focus on detailed profiles of inclisiran, mainly its delivery strategies. We discuss the mechanisms of action, its status in clinical trials, and its prospects.

## 1. Introduction

The different origins and structures of small non-coding RNAs have led to the identification of three main categories: piwi-interacting RNA (piRNAs), small interfering RNA (siRNAs), and microRNAs (miRNAs). Development of siRNA molecules is reasonably undemanding once a target mRNA sequence has been determined; hence it is a popular tool for controlling gene expression. siRNA is a double-stranded RNA molecule, which is non-coding, short and well-defined (~21–25 base pairs long), with hydroxylated 3′ and phosphorylated 5′ ends. Since the first discovery of siRNA and their role in post-transcriptional gene silencing (PTGS) in plants, it has played a significant role in many functional studies for the post-human-genome era. In the early 2000s, it was anticipated that knowledge of the human proteome would identify more than 3000 potentially druggable proteins and 600–1500 promising small-molecule drug targets [1,2,3,4,5,6]. Conventional small molecules acting as therapeutic agents function at the protein level, necessitating a higher structural accuracy and, thus, a more complex and challenging developmental process [7]. Among the many limitations, one must consider various side effects, early drug elimination, non-specific cytotoxicity, high dosing levels, multi-drug resistance, and low bioavailability. Monoclonal antibodies have emerged as the leading class of therapeutic agents in the treatment of numerous human diseases, such as cancers, immunological, infectious (such as cytomegalovirus, hepatitis A and B viruses) [8], and metabolic disorders. The market for monoclonal antibodies has experienced exponential growth in recent years, even though therapeutic monoclonal antibodies are restricted to binding with cell surface receptors or circulating proteins. As a result, finding and developing alternative therapeutic approaches, such as RNA techniques, is an ongoing quest for academic research chemists and pharmaceutical companies. 

Small interfering RNAs (siRNAs) enable gene-targeted silencing. This compromises gene expression and regulation by cleaving mRNA or repressing its translation [9]. Effective knockdowns of disease-associated genes with siRNAs open the door for new therapeutic techniques, such as chemical modifications of siRNA and direct and indirect mutation targeting, among others [10,11,12]. siRNA technology faces many barriers. The first challenge is overcoming several intracellular and extracellular defenses designed to prevent exogenous RNA from entering cells [13,14,15,16]. More generally, in the systematic administration of siRNA, many physiological barriers must be overcome to achieve the following: (a) the crossing of the vascular endothelium to reach target tissues, (b) nuclease stability, (c) delivery to the specified target, (d) renal filtration, (e) reaching the cytoplasm of the target cells, and (f) incorporation into the RNA interference machinery [13,17,18,19,20,21,22,23,24,25]. Strategies for intracellular administration of siRNA are expected to be non-toxic and to have good stability at the site of action [17,26]. Advances in the distribution of siRNA drugs continue as investigators maximize the specificity of siRNA administration while lowering the associated toxicity and degradation effects that reduce drug effectiveness [27]. There are two distinct approaches that enable the delivery of siRNAs to target tissues that have been reported in the literature: lipid nanoparticles (LNPs) and conjugates [28]. For example, patisiran (Onpattro), an RNAi therapeutic drug that uses LNP-based delivery. Patisiran may not be used in combination with additional ribonucleic acid interfering drugs or transthyretin stabilizers that are used to treat hereditary transthyretin-mediated amyloidosis (hATTR). Further, siRNAs are becoming novel nucleic acid drugs for undruggable targets to manage terminal illnesses such as cancers [29,30]. However, due to the systemic administration that is needed in most incidents, there are vital challenges in developing siRNAs for cancer therapies [31]. Givosiran was approved to treat acute hepatic porphyria (AHP) in adults [32]. Vutrisiran can treat hATTR in adult patients with polyneuropathy. At the end of 2021, Novartis announced the US Food and Drug Administration (FDA) approval of a new drug, inclisiran (Leqvio), as the first and thus-far only siRNA therapy that decreases low-density lipoprotein cholesterol (bad cholesterol or LDL-C) [33,34]. Notably, inclisiran is a drug that is produced using *N*-acetylgalactosamine (GalNAc) conjugates (see Table 1).

## 2. Lipid Nanoparticles (LNPs) and GalNAc Conjugates for siRNA Delivery

### 2.1. Lipid Nanoparticles (LNPs)-siRNA

**Figure 1:**
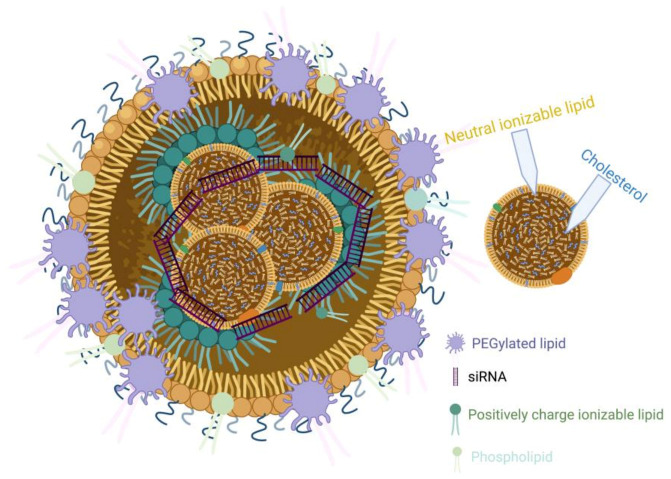
Schematic illustration of LNPs containing siRNA and other key lipid components.

LNPs have appeared primarily as a preferred carrier to transport a variety of therapeutic agents. They are clinically promising to enhance nonviral and FDA-approved nanomedicines and to deliver siRNAs to a specific target [35,36]. LNPs have many pros, such as formulation simplicity, biodegradability, biocompatibility, high bioavailability, the ability to transport large payloads, and a series of physicochemical properties that can be structured to regulate their biological features [37,38,39,40,41]. Enroute to their terminus, siRNAs that are encapsulated in LNPs are effectively protected against breaking down by ubiquitous nucleases. Degradation is delayed, thus promoting the drug’s efficacy and presenting long-lasting effects for any target. Ionizable lipids are protonated at a low pH, which makes them positively charged, but they remain uncharged under physiological conditions to impede rapid sequestering by immune cells during systemic circulation (positive nanoparticles are quickly segregated by Kupffer cells in the liver and splenic macrophages) [25,42,43,44,45]. LNPs can be loaded with nucleic acid polymers at pH values lower than pKa of the ionizable lipid, which is positively charged. Sensitivity to the pH of ionizable lipids significantly influences the administration of mRNA in vivo, as neutral lipids have a reduced association with the anionic membranes of blood and cells, thereby increasing the biocompatibility of LNPs [46,47,48,49,50]. Notably, the pKa of an ionizable lipid should be sufficiently low for it to remain unprotonated during circulation but reasonably high to be protonated in the initial or deferred endosome to maintain efficacy and reduce toxicity [21]. Currently, lipids, cationic ionizable lipid, cholesterol, phosphatidylcholine, and a poly(ethylene glycol) (PEG) lipid (Figure 1) are the critical components for formulations. Also, the effective delivery of siRNA in LNPs has demonstrated favorable outcomes in different phases of clinical studies. 

#### 2.1.1. Ionizable Cationic Lipids

**Figure 2:**
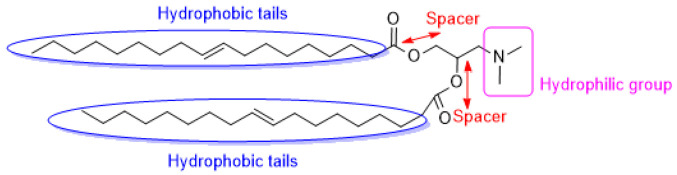
Chemical structure of DODAP, a cationic ionizable lipid.

The ionizable cationic lipid has been enhanced for RNA encapsulation and facilitates delivery [51,52]. Cationic lipids that are used for gene therapy are composed of three basic domains: a positively charged group, a hydrophobic tail, and an amine spacer to bridge the polar and non-polar motif. A sequence of ionizable lipids has been designed and synthesized for siRNA delivery, with specific pKa and physical properties. Namely, 1,2-dioleoyl-3-dimethylaminopropane (DODAP), 1,2-dilinoleoyl-3-dimethylaminopropane, 1,2-dilinoleyloxy-3-dimethylaminopropane (DLin-DMA), 2,2-dilinoleyl-4-dimethylaminomethyl -dioxolane (DLin-KC2-DMA), 2,2-dilinoleyl-4-(2-dimethylaminoethyl)-dioxolane (DLin-KC2-DMA), and (6Z,9Z,28Z,31Z)-heptatriaconta-6,9,28,31-tetraen-19-yl-4-(dimethylamino)-butanoate (Dlin-MC3-DMA) with pKa values of 6.6, 6.8, 5.9, and 6.4, respectively (Table 2). DODAP is the first example of an ionizable lipid, and rapid mixing with other lipids such as PEG-lipid, cholesterol, and distearoylphosphatidylcholine (DSPC) and oligonucleotides in ethanol allowed high encapsulation efficiencies [53,54]. Meanwhile, DLinDMA is the first ionizable lipid that caused substantial hepatocyte gene silencing in vivo; the formulations demonstrated minimal effectiveness with high doses of siRNA [55]. Despite the efficacy of DODAP and prolonged hepatocyte knockdown using DLin-DMA, they never succeeded in extending studies to the clinical trial. This may be due to their unacceptable toxicity, poor tolerability and potency, and unspecific association to negatively charged cellular and extracellular components. Figure 2 displays the three basic domains of DODAP. The polar and hydrophobic tails of the cationic lipids are an essential component resulting in favorable effects on both the transfection and toxicity of the lipid. 

Moreover, the variation of the spacer is an essential factor to consider for delivery efficiency. The introduction of a ketal linker significantly influences the delivery efficacy with good in vivo activity. Reduced efficacy is observed when the ketal is substituted with the ester or alkoxy functional group [60]. In designing a new library of ionizable lipids and increasing the delivery system’s efficacy, the linker and the hydrophobic regions of DLinDMA were modified, resulting in the identification of DLin-KC2-DMA. Meanwhile, modifying the amine functional group led to the production of DLin-MC3-DMA. Jayaraman and co-workers reported that Dlin-MC3-DMA (ED50~0.03 mg/kg) was 10-fold superior compared to Dlin-KC2-DMA (ED50~0.3 mg/kg) for hepatic gene silencing in vivo [51,57]. DLin-MC3-DMA is currently the most promising cationic lipid for therapeutic genetic drug carriers that is approved by the FDA and a fundamental delivery constituent of Onpattro, the first FDA-approved siRNA drug. It can be concluded that the significant factors influencing cationic lipid power include acyl chain unsaturation, ether bonds, and the pKa of the cationic lipid amino headgroup [61].

#### 2.1.2. Poly (Ethylene Glycol) (PEG)

**Figure 3:**
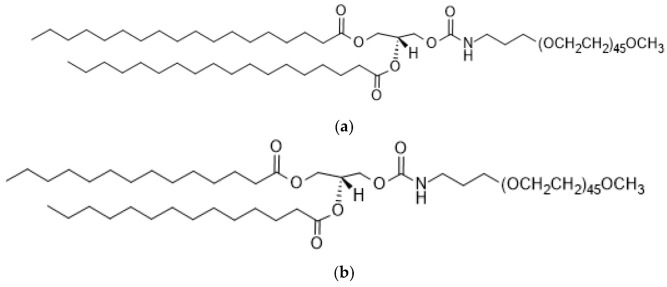
Chemical structure of PEG-lipids, (**a**) DMG-PEG2000 and (**b**) DSG-PEG2000. (**a**) 1,2-dimyristoyl-rac-glycero-3-methoxypolyethylene glycol-2000 (DMG-PEG 2000). (**b**) 1,2-distearoyl- rac-glycero-3-methoxypolyethylene glycol-2000 (DSG-PEG 2000).

PEG-lipids can have multiple effects on the properties of LNP [43,62,63,64,65] and PEG-lipids have been engineered to control LNP size and produce higher transfection efficiencies [66,67]. In addition, the type and amount of PEG-lipid utilized are proportional to the size, and this significantly affects the gene-silencing potency of LNP siRNA systems [68,69]. Further, it provides drugs with more significant physical and thermal stability and inhibits drug aggregation in vivo [63]. PEG-lipids such as 1,2-dimyristoyl-rac-glycero-3-methoxypolyethylene glycol-2000 (DMG-PEG2000) and 1,2-distearoyl-rac-glycero-3-methoxypolyethylene glycol-2000 (DSG-PEG2000) are uncharged with an alkyl chain length of C_14_ and C_18_, respectively (Figure 3). In addition, the length of lipid chains that are attached to the PEG affects how long it stays on liposomes. DSG-PEG2000 demonstrated longer retention times compared to DMG-PEG2000. PEG lipids with short (C_14_) lipid tails tend to break free from LNP-siRNA in vivo with about a half-time of 1 h in LNPs–siRNA formulations. However, PEG lipids with increased (C_18_) alkyl chains display dissociation rates of days or longer [50,70]. Increasing the number of carbon chains did not satisfactorily change the hepatic gene silencing, delivery efficacy, and pharmacokinetic features of LNPs [55,69,71]. This suggests the likelihood that LNP stabilization by PEG lipids is a functionality of short alkyl chains that quickly desorb after application which may be advantageous for LNP cellular uptake and endosomal escape [64,65,72,73,74]. 

#### 2.1.3. Cholesterol and Saturated Phosphatidylcholine (PC)

Liposomes comprise one or several lipid bilayers, between 20 and ~1000 nm in size, that serve as suitable delivery cargo for therapeutic agents [38,75,76]. Liposomes can encapsulate hydrophobic and hydrophilic agents in the lipid membrane and the aqueous core. In contrast, the hydrophilic fragment can defend the loaded drugs from the host bodies’ damaging features that eventually decrease the undesirable side effects [77,78,79,80,81,82,83,84,85]. The development of liposomes as a drug carrier system has been advanced in the last few years to improve pharmacokinetics. Their capacity to deposit their cargo enhances drug efficacy and minimizes side effects [74,80]. Cholesterol and saturated phosphatidylcholines (PC), such as distearoyl-PC(DSPC) or dipalmitoyl-PC (DPPC), are commonly employed liposome substituents that favorably influence the strength of the LNP formulation. However, inadequate evidence obscures the purpose behind the use of cholesterol from the perspective of nucleic acid delivery systems. A mixture of unsaturated phospholipids in a lipid delivery system was reported to enhance the capacity of the phosphor-lipid membrane to form a stable bicontinuous cubic phase at physiological conditions, hence improving fusogenicity [86]. In particular, the development of inverted bi-continual cubic phases is directly related to lipid membrane fusion [86,87]. Notably, there must be a focus on critical issues such as cost-effectiveness, off-target effects, and toxicity as we advance, despite the rapid silencing, expression, or modification of genes in human patients using LNPs technology. Based on the understanding that is gained through the design, development, and use of the first FDA-approved and marketed LNP−siRNA drug, the technology can be improved for future applications [55].

### 2.2. N-Acetylgalactosamine (GalNAc)-siRNA

Asialoglycoprotein receptor (ASGPR), also known as hepatic binding protein or the Ashwell–Morell is a C-type lectin and the first animal lectin that was identified during turnover studies of a serum glycoprotein, ceruloplasmin [88,89,90,91,92,93]. Several studies have shown hepatic lectin as a promising candidate target as a drug and gene carrier into hepatocytes [94,95]. ASGPR is comprised of two homologous units: a significant subunit (ASGPR-1) 48 kDa and a minor 40 kDa subunit (ASGPR-2). This heterooligomeric receptor is amply articulated on the sinusoidal (i.e., basolateral) surface of the liver hepatocytes [96,97,98,99,100,101,102]. ASGPR mediates binding and internalization, followed by clearance of glycoproteins enfolding terminal galactose or *N*-acetylgalactosamine (GalNAc) residues from the circulation [91,103]. It has been reported that in the past three decades, asialoglycoprotein, galactoside, galactosamine, galactan, and galactose derivatives, namely GalNac, have been extensively studied to deliver naturally active glycopeptides [104], antisense peptide nucleic acid (asPNA) [105], glycolipids [106], small molecules [107], nucleoside analogues [108,109], plasma DNA [110,111], and ASOs [112,113,114] to the central functional parenchymal tissue of the liver. Also, a molecular hybrid of siRNA with GalNAc hastens the uptake of hepatocytes by binding with the asialoglycoprotein receptor (ASGPR) [115,116]. Patisiran is used for treating hereditary TTR amyloidosis and it utilizes LNP for liver delivery. However, due to the intricate formulation of LNP and the challenge in administration routes, GalNAc technology, which is uncomplicated, smaller, and with a distinct formation methodology, is currently emerging and superseding LNP as a delivery vehicle of small interfering RNA (siRNA) to target tissues. 

**Figure 4:**
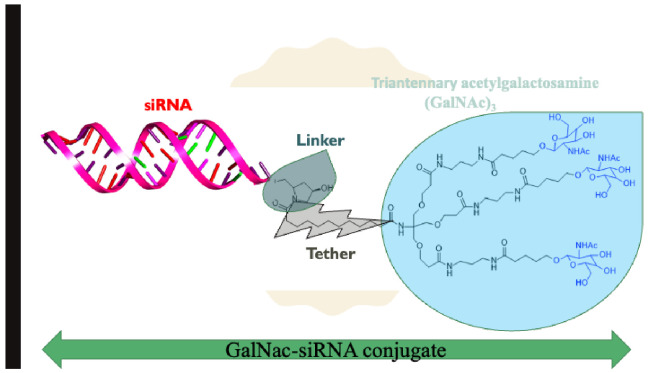
Chemical structure of triantennary acetylgalactosamine (GalNAc)_3_ hybridized with siRNA.

GalNAc-siRNA and GalNAc-ASO (antisense oligonucleotides) conjugate delivery studies have shown exquisite superiority overall for this delivery approach. ASOs are short, chemically-modified oligonucleotides that bind to the RNA in cells via Watson–Crick base-pairing and modulate RNA function to cause a pharmacological effect [117]. The Watson–Crick molecular detection provides the antisense field more flexibility in RNA-based drug design. It accelerates development and is vital for targeting a myriad of rare and genetic diseases [118,119]. Covalently linked ASO-triantennary GalNAc, was shown to provide favorable gene silencing [113]. The ED_50_ of GalNAc conjugated 5-10-5 MOE gapmer targeting SRB1 mRNA in mouse liver was 3.3 mg/kg and superior to unconjugated gapmer ASO with ED_50_ of 18.3 mg/kg [120]. The replacement of the MOE nucleotide sugar wings in gapmer ASO with constrained ethyl (cEt)-bridged ribonucleotides led to a ~60-fold augmentation in relation to the parent MOE (2′-*O*-methoxyethyl RNA) [113]. The amalgamation of three GalNAc moieties to one siRNA with a functional spacer is shown in Figure 4. The methodology favors the high affinity of the interaction between ASGPR and the GalNAc ligand and the optimal efficiency of siRNA delivery to hepatocytes via subcutaneous injection. Further, the enhanced stabilization alteration allows the amalgamation of GalNAc- siRNA to remain in the circulation system and cytoplasm for more than 100 days [121]. Remarkably, this enables long-term gene silencing and therapeutic effects in humans after a single dose [122,123,124]. 

**Figure 5:**
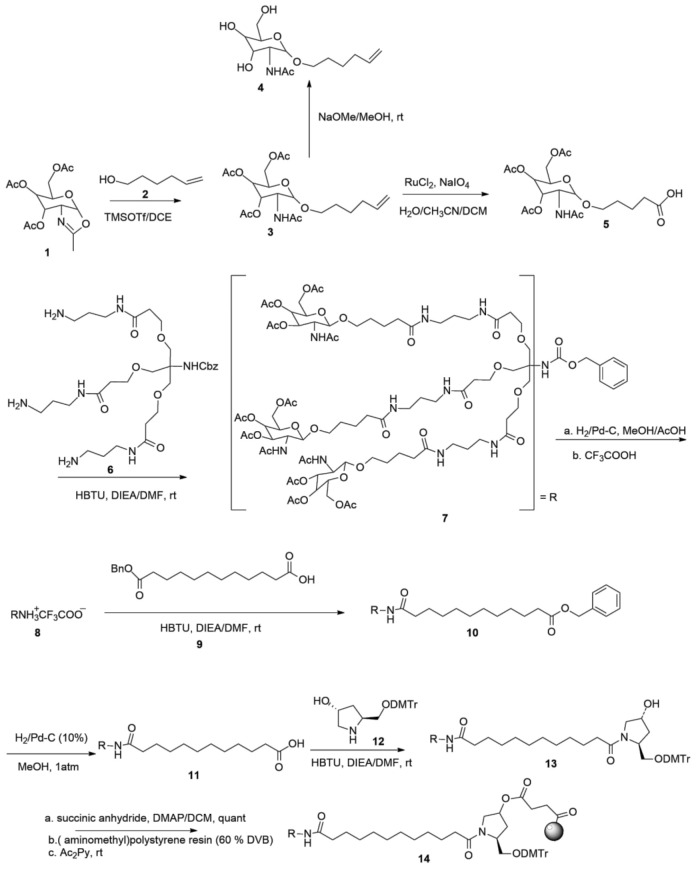
Synthetic route to triantennary acetylgalactosamine (GalNAc)_3_.

A synthetic route to GalNAc conjugates has been obtained together with a method to covalently link GalNAc to siRNAs [116]. GalNAc-conjugated siRNAs confer high-affinity binding to the ASGPRs, which is highly efficient, hepatocyte-specific, and clathrin-mediated endocytic. Trivalent GalNAc clusters have higher affinity, efficacy, and apparent advantages than divalent, trivalent, and tetravalent galactose-based conjugates [95,116,125,126]. The GalNAc_3_ arranged in the triantennary approach, as shown in Figure 5, was found to be the most favorable chemically improved and metabolically stable GalNAc conjugates for ASGPR-mediated siRNA uptake. It is a long-acting treatment for different diseases relating to liver-expressed genes [116]. In mice, the TTR gene was silenced in the liver with a single-dose median effective dose (ED_50)_ of ∼1 mg/kg subcutaneously). Chronic weekly medication of GalNAc-siRNA conjugate resulted in continued dose-dependent gene silencing for >9 months with no after-effects in mice. Alnylam Pharmaceuticals uses triantennary GalNAc-siRNA conjugates as a delivery vehicle, and they have designed and synthesized more than five novel GalNAc-siRNA hybrids to target different diseases. For example, givosiran is a GalNAc-conjugated siRNA therapeutic agent targeting 5′-aminolevulinate synthase 1 (*ALAS1*) that is approved for the treatment of acute hepatic porphyria (AHP) in the United States and in the European Union [127]. 

Inclisiran is also an approved GalNAc-conjugated siRNA containing 2′-F, 2′-OMe and PS modifications. As mentioned above, inclisiran is used in a specified maximum-tolerated statin therapy for adults with ASCVD or heterozygous familial hypercholesterolemia (HeFH), which require further lowering of LDL-C. Meanwhile, CVD is one of the leading global causes of mortality. In order to reduce the death rate from CVD, the U.K.’s National Health Service (NHS) has recommended the use of inclisiran to treat patients with ASCVD [128].

## 3. Inclisiran

### 3.1. Some Selected Cholesterol-Lowering Drugs before Approval of Inclisiran

Hypercholesterolemia is a leading risk factor for CVD. A high concentration of low-density lipoprotein cholesterol (LDL-C) is associated with a greater risk of heart attack, stroke, and ensuing complications [129]. The reduction of LDL-C has been clearly shown to lead to a substantial decrease in CVD incidence. Conventional drugs that are used to reduce high cholesterol before approval of inclisiran include statins, niacin, bile acid drugs, fibrates, and antibodies acting as PCSK9 inhibitors. Statins were the first widely used high-cholesterol therapies (for example, lipitor, lescol, pravachol, crestor, and zocor). They prevent hardening of the arteries, decrease the risk of heart attacks or strokes, and enhance the reduction of LDL by obstructing vital enzymes that are imperative for cholesterol assembly. Niacin (niaspan and nicoar), a vitamin B, is orally administered in appropriate concentrations for therapeutic applications. Niacin can effectively lower LDL and has benefits in raising high-density lipoprotein (HDL) cholesterol and lowering triglyceride (TG) levels and is often used as a combination therapy with statins [130,131,132]. Fenofibrate and gemfibrozil are fibrates that reduce the production of triglycerides. Both treatments exert their effects on HDL through peroxisome proliferator-activated receptor (PPARα) [133,134]. Bile acid acts by binding to the bile of the liver. Examples of approved bile acid sequestrants include cholestyramine (questran), colestipol (colestid), and colesevelam. Colesevelam has superior binding and affinity for biliary acids than cholestyramine and colestipol. It can be administered at considerably lower doses to obtain minimal side effects [135]. Notably, the reduction of the LDL-C level depends on the specific drug and the quantity that is administered; bile acid sequestrants can be administered as monotherapy (10% reduction of LDL-C) or as combination therapy with statins (25% reduction of LDL-C) [136,137]. Ezetimibe (zetia) as a combination therapy with a statin can successfully impede cholesterol absorption, leading to a reduction in LDL-C levels without interfering with the absorption of triglycerides, fatty acids, bile acids, or fat-soluble vitamins such as vitamin D and K [138]. PCSK9 inhibitors were first approved in 2015 and are more effective in lowering serum cholesterol levels than statins [139]. These inhibitors act by binding to PCSK9 with high affinity, deactivating PCSK9 in the blood and interfering with its ability to reduce LDL cholesterol (bad cholesterol) uptake by hepatocytes—all without apparent negative health consequences. Notably, the combination of a PCSK9 inhibitor and a (low dose) statin may further increase the lipid-lowering effects while preventing the side effects [140,141,142,143].

PCSK9 was initially known as neural apoptosis-regulated convertase 1 because levels of this protein are high in cerebral neurons during apoptosis [144,145,146]. It was subsequently discovered that PCSK9 loss-of-function modifications were clinically significant and cause dominant hypocholesterolemia while reducing CVD risk. This is because PCSK9 binds to (and degrades) the LDL-C receptor at the surface of hepatocytes, thus impeding LDL uptake from blood [147,148,149]. Monoclonal antibodies against PCSK9 are currently available: alirocumab (praluent) and evolocumab (repatha). Evolocumab was FDA approved for adults with HeFH including patients with significant atherosclerotic CVD who require further reduction of LDL C after undergoing a controlled diet and maximum tolerated statin therapy. Alirocumab and evolocumab, either in monotherapy or in combination with lipid-lowering therapies, significantly reduced LDL-C levels by up to 60% in patients with high cholesterol. Such as those with familial hypercholesterolemia, moderate to very high cardiovascular risk, and statin intolerance [149,150,151,152,153,154,155]. 

Most of these drugs have proven effective in lowering high cholesterol and decreasing mortalities and complications from CVD. However, the adverse effects of these drugs cannot be overlooked. For example, despite optimal statin therapy or statin intolerance, many patients fail to achieve LDL-C treatment targets [156]. Statins and fibrates have been reported to cause severe myopathy [157,158]. The drug interactions involving statins result from inducing or inhibiting different CYP isoenzymes leading to an increase in the risk of myopathy, rhabdomyolysis, diarrhea, joint pain, fatigue, and other severe adverse events [159]. Niacin is effective in the treatment of children with hypercholesterolemia [160]. The adverse effects include muscle pain, liver problem, tiredness or weakness, loss of appetite, upper stomach pain, and itching. PCSK9 blockers, administered once or twice a month, reduce circulating PCSK9 levels and decrease LDL cholesterol levels without severe adverse effects. Long-term therapy has a lower incidence of CVD than controls. However, PCSK9 antibodies have a short effect interval, requiring recurrent intravenous injections. Hence, there is a necessity for additional therapy that brings the possibility of lowering LDL cholesterol levels with minimal adverse effects. 

### 3.2. Development of Inclisiran

**Figure 6:**
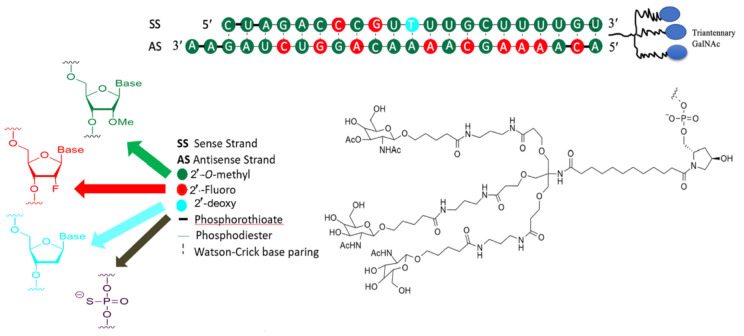
Chemical structure of inclisiran.

Inclisiran (Figure 6) is a chemically-modified small interfering RNA (siRNA) molecule that is conjugated to GalNAc to target PCSK9. It is the first siRNA therapeutic class across the medical spectrum to be subjected to exhaustive assessment in clinical trials to determine its efficacy in reducing LDL-C and precluding CVD. Inclisiran sodium has a molecular formula of C_529_H_664_F_12_N_176_Na_43_O_316_P_43_S_6_ and a molecular weight of 17,284.75 g/mol, both parameters in salt form. Inclisiran sodium was formed by synthesizing two single-strand oligonucleotides, sense and antisense strands, by exploring the established solid-phase approach [161]. The 3′-*O*-(2-cyanoethyl) phosphoramidite with the 5′-hydroxyls protected with 4,4′--dimethoxytriphenylmethyl (DMT) moiety and fluoro-, OMe-, or deoxy-changes at the 2′-position was utilized. However, the sense constituent is synthesized on protected GalNAc-based ligand such as L96-loaded polymer support and the antisense constituent on 2′-OMe A-loaded controlled pore glass (CPG) support. The sense strand is coupled with GalNAc to accelerate fast hepatocyte uptake with high specificity only by the liver after subcutaneous low-volume injection. The sense strand consists of 21 and 23 nucleotides in the antisense strand. The single-strand oligonucleotides are cleaved from the solid support, deprotected, and subsequently ultrafiltrated, followed by HPLC to eliminate impurities. Purification was also performed after the second ultrafiltration to eliminate the substances from the mobile phases that are used in chromatography. Afterward, the two single strands were mixed in an equimolar ratio during annealing, followed by concentration and freeze-drying [161]. The chemical structure of inclisiran consists of 1 2′-deoxy, 11 2′-fluoro-, and 32 2′-O-methyl-modified nucleotides, and a triantennary N-acetylgalactosamine (GalNAc) that coupled with the 3′-end of the passenger strand. 

Since unmodified siRNAs lack stability because of the high susceptibility of ribonucleotides to exonuclease, they are susceptible to degradation in serum [162,163,164]. There are five alterations that are utilized, or more specifically, phosphorothioate, 2′-deoxy, 2′-fluoro-RNA, 2′-*O*-methyl-RNA, and triantennary GalNAc–all without loss of efficacy. The amalgamation of the 2-fluoro and 2-*O*-methyl changes permits a significant stabilization of inclisiran without obstructing the siRNAs from immediately entering the RNA-induced silencer complex (RISC) [165]. The RISC protein is crucial for refining transcriptional profiles in cells, improving the stabilization of siRNAs. The terminal phosphodiester bonds are improved with phosphorothioates and provide further stabilization against exonuclease degradation and potentially influence the cellular internalization of the compound. It is worth pointing out that modifying 2′-fluoro and 2′-O-methyl and the additional four phosphorothioates to the inclisiran are liable for lessening the degradation of the molecule in the bloodstream [166].

### 3.3. Clinical Trials of Inclisiran

The ALN-PCS molecule, a precursor to inclisiran, was incorporated into a lipid nanoparticle. It decreased PCSK9 mRNA and protein concentrations by 70% and 60% in LDL-C concentrations over 3 weeks in an in vivo study [167]. The molecule caused a 70% decrease in PCSK9 protein after a 60-minute infusion and a 40% decrease in LDL-C levels on the third day of follow-up in a clinical trial [168]. The ALN-PCS molecule was later introduced by *N*-acetylgalactosamine (GalNAc) to the molecule to produce ALN-PCSSC. This improved its clinical efficacy and extent of action by increasing the uptake of the hepatocyte cell membrane and liver cell specificity. The results of clinical studies of ALN-PCS (Table 3) [169] suggest that inhibiting PCSK9 synthesis through RNA interference (RNAi) gives an effective, safe mechanism for lowering the concentration of LDL cholesterol in healthy patients with elevated cholesterol. These findings support further evaluation of ALN-PCS in individuals with hypercholesterolemia, comprising those receiving statin therapy. This first report shows the drastic influence of RNAi drugs on the clinically confirmed endpoint (i.e., LDL-C) in humans. Liver function tests, troponin, or inflammatory markers (cytokines and C-reactive protein) had no clinically significant changes. 

A multiple-dose study to evaluate the safety, tolerability, pharmacokinetics, and pharmacodynamics of subcutaneously administered ALN PCSSC in subjects with elevated LDL cholesterol was then conducted [124]. The results of this study demonstrated clinical evidence for the use of PCSK9 as a therapeutic target for a significant reduction in LDL cholesterol, which can be effective for at least six months after starting treatment. This is highly attractive when compared to the endorsed regimens of PCSK9 antibodies that are administered once or twice monthly. Notably, inclisiran is different from anti-PCSK9 antibodies. Anti-PCSK9 antibodies attach to extracellular PCSK9 and block it from interacting with the LDL receptor, while inclisiran impedes the production of the PCSK9 protein precisely in the liver. Secondly, the pharmacodynamic properties of inclisiran differ significantly from that of anti-PCSK9 antibodies. 

ORION-1 was a Phase 2, placebo-controlled, double-blind, randomized trial of volunteer participants with ASCVD or ASCVD-risk equivalents such as diabetes and FH as well as participants with elevated LDL-C, despite a maximum tolerated dose of LDL-C lowering therapies. This is to measure the efficacy, safety, and tolerability of ALN-PCSSC injection(s) [170]. The results showed that PCSK9 levels were decreased from baseline levels by a mean of 59.6 to 68.7% throughout the range of inclisiran doses from 100 mg to 500 mg after 14 days of a single injection. Further, at day 180, average reductions from reference concentrations of PCSK9 in patients on a two-dose diet ranged from 53.2% to 69.1%. The most common adverse events in inclisiran-treated and placebo-treated individuals (>2% of patients) were myalgia, headache, fatigue, nasopharyngitis, back pain, hypertension, diarrhea, and dizziness [171]. Immune activation, typically found in RNA therapies, was occasionally associated with inclisiran. There have been flu-like symptoms but no observed elevated C-reactive protein.

The ORION-3, open-label, non-randomized, active comparator extension trial was registered to assess the efficacy, safety, and tolerability of long-term dosing of inclisiran and evolocumab given as subcutaneous injections in participants with high cardiovascular risk and elevated low-density lipoprotein cholesterol (LDL-C). On day 210 of the ORION-3 test, LDL-C concentrations were reduced by an average of 51%, while PCSK9 concentrations were reduced by 77%. A long-term effect of 300 mg of inclisiran on lowering LDL-C was observed in ORION-3 for approximately 22 months. Further, the LDL-C was reduced over time by about 60 mg/dL. Meanwhile, no changes were made to the overall safety profile for a minimum of three years of monitoring. No laboratory abnormalities that were associated with therapy, including liver and renal function tests, were observed since the successful study of the Phase 2 clinical trial. The Phase 3 trial focused on evaluating the use of inclisiran in a large group of adult participants with HeFH who had been treated with a maximally accepted dose of statin therapy.

A total of three Phase 3 studies, i.e., the ORION-9 (NCT03397121), ORION-10 (NCT03399370), and the ORION-11 (NCT03400800) trial, two randomized, double-blind, placebo-controlled, parallel-group, were registered [123,172,173]. This was done to determine the efficacy, safety, and adverse-event profile of inclisiran over 18 months in patients, either at elevated or excessive CV risk with an LDL-C ≥ 70 mg/dl (in a patient with ASCVD) or ≥100 mg/dl (in patients with an ASCVD risk equivalent). Even though the patients are given statin therapy at the maximal dose tolerated with or without ezetimibe, the results of the analysis identified 284 mg of inclisiran exhibited statistically considerable developments compared to placebo by reducing LDL-C concentrations in adult patients with HeFH or nFH with ASCVD who received the maximum tolerated dose of a statin or who were statin intolerant. The cluster variances in percentage change in LDL-C from baseline to day 510 were −49.52 in ORION-9; −57.64 in ORION-10, and −53.5 in ORION-11 (all *p* < 0.0001). Adverse occurrences that were reported for ORION-9 were 76.8% in the inclisiran group and 71.7% in the placebo group. Many reported occurrences were mild or moderate. A greater number of patients in the inclisiran group than in the placebo group reacted at the protocol-defined injection site, at 17.0% compared to 1.7%. In the ORION-9 trial, 90.2% of events were classified as mild, and none were reported as severe or persistent. The number of severe adverse events that were associated with inclisiran was lower than those that were associated with the placebo (7.5% compared to 13.8%). Lowering LDL-C levels in circulating plasma reduces CVD risk. This will improve CVD outcomes because high cholesterol is an important risk factor for cardiovascular disease.

### 3.4. Inclisiran-Mechanism of Action

**Figure 7:**
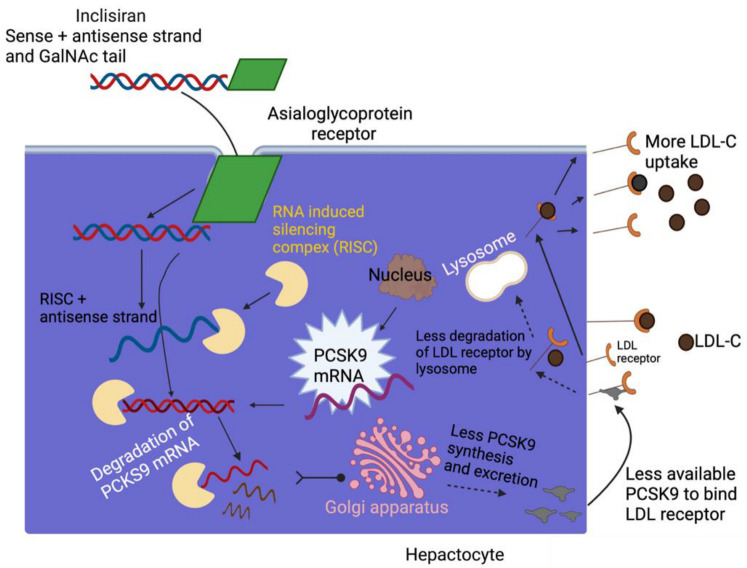
Schematic of the mechanism of action of inclisiran. Figure created with BioRender.com (accessed on September 29, 2022). Adapted from Cupido AJ, Kastelein JJP. *Cardiovasc Res*. 2020;116(11):e136-e139. GalNAc—N-acetylgalactosamine; LDL—low-density lipoprotein; LDL-C—LDL cholesterol; LDL-R—LDL receptor; PCSK9—proprotein convertase subtilisin/kexin type 9; RISC, RNA—induced silencing complex; siRNA—small interfering RNA.

Inclisiran is a chemically synthesized siRNA that is conjugated with carbohydrates N-acetylgalactosamine triantennary and not only interacts but binds to ASGPR, a specific liver receptor. This promotes the absorption of inclisiran in hepatocytes. ASGPR is a transmembrane protein that is composed of two subunits, a significant subunit, ASGR1, and a minor subunit, ASGR2, expressed primarily through hepatocytes [174]. It has been shown that ASGR1 expression is wholly associated with protein levels in the liver, which could affect drug administration by ASGPR. [175]. In particular, the ASGR1 subunit was found to be the most severely implicated sub-unit. Human models using variations in ASGR1 may be used to evaluate the ability of ASGPR to administer drugs [176,177]. Subsequently, inclisiran binds with the cytosolic RISC, a ribonucleoprotein complex that acts as a model for identifying the complementary target mRNA. Thus, the activated RNAse cleaves the target mRNA, which ejects one strand, exiting the antisense strand to bind to the target mRNA and facilitate its sequence-specific cleavage by the RISC RNase Argonaute. As the incorporation of inclisiran RISC tolerates the drug to bind to the mRNA PCSK9, it further obstructs the translation of PCSK9, and hence the degradation of hepatic production of PCSK9. Meanwhile, the lower the synthesizing of PCSK9 protein, the more the recycling of LDL receptors, thus causing a reduction in the levels of LDL-C in the blood. In summary, inclisiran binds to the precursor of the mRNA of PCSK9, inhibiting its translation and production of PCSK9, and subsequent PCSK9 protein reduction promotes LDLR recycling, increasing absorption and degradation of plasma LDL-C and decreasing plasma LDL-C levels [177,178,179] (see Figure 7).

### 3.5. Clinical Benefits of Inclisiran

Administration of inclisiran twice a year has been established to significantly improve treatment adherence and clinical efficacy, such as lowering the level of LDL-C for a more extended period. This is an undeniable benefit of inclisiran compared to other drugs that lower LDL-C. Most require daily administration, increasing the likelihood of poor adherence to medical treatment, which severely compromises optimal outcomes, leading to high rates of withdrawal from treatment, reduced achievement of LDL-C targets, and lesser medical effects [180]. Further, an efficient and continuous decrease can be afforded using inclisiran for LDL-C levels up to 52% compared with placebo for some patients with ASCVD on treatment with statins to the maximum tolerated [34,123]. 

## 4. Other Critical Facts on siRNA Therapeutic and Inclisiran

RNA technology has been able to resolve the issues of stability, delivery system, translation efficiency, and immunogenicity to accomplish effective, safe, and non-toxic delivery of siRNA. However, despite the significant improvement of the inclisiran therapy in treating high cholesterol and inhibiting CV events, some questions remain unanswered. The review of the age group of volunteers who took part in the completed clinical trial on inclisiran showed that it is not yet known if inclisiran is safe enough to be used in young patients with homozygous family hypercholesterolemia (HoFH). However, the worldwide Phase III placebo-controlled trials ORION-13 and ORION-16 are currently enrolling patients to evaluate and determine the short-term effectiveness, safety, and tolerability of inclisiran in adolescents with HoFH and HeFH, respectively, and high LDL-C on the stable standard of hypolipidemia therapy. This could be one of the drawbacks of inclisiran if this current study does not answer these issues. Furthermore, there is still a lack of long-term data on participants who have experienced major adverse cardiovascular events (MACE). There are two current trials that are registered to examine the effects of inclisiran on major adverse cardiovascular events (ORION-4 and VICTORION-2P). The present VICTORION-2P study aims to assess that inclisiran sodium therapy should be administered on day 1, day 90, and every six months (30 mg). After that, in addition to statin therapy in patients with ASCVD, the risk of CV mortality, non-fatal myocardial infarction (MI), and non-fatal ischemic stroke is substantially lessened. This is comparable to placebo in adjuvant to well-tolerated high-intensity statin therapy. At the same time, ORION-4 trials aim to determine if inclisiran will safely reduce the risk of a heart attack, including strokes, in individuals who have already had any of these conditions or who have had an operation or procedure to treat clogged arteries. However, some uncertainty remains whether treatment with inclisiran will translate into a decrease in MACE rates that are akin to when statins or PCSK9 inhibitors are used. Also, long-term observation of a drug is critical because some potential side effects may not have had time to occur. The semi-annual administration of inclisiran may impact the patient’s desire to take the medication for longer-term therapy. Although this hypothesis has not yet been proven, a long-term extension of Phase III (ORION 8) has been registered to appraise the consequence of long-term dosing of inclisiran in subjects with high CV risk and raised LDL-C (ORION-8). This will strengthen confidence related to the safety profile of inclisiran therapy. This also indicates that opportunities for improvement and novelties cannot be underestimated. Thus, several dominating companies in the RNA biopharma sector will become apparent, and new small biotech start-ups and academic groups with transformative concepts will continue to propagate [181]. Some of the challenges in the siRNA therapeutic still need urgent attention by providing a better and more efficient delivery vehicle, regulation of the activated immune system, structure-based antigen design, and delivery system optimization to enhance stability and degradation [182]. The modification or optimization of the siRNA delivery strategies can support transporting siRNAs to a wide range of targets. Notably, GalNAc-siRNA conjugates have considerably higher plasma clearance due to more efficient hepatocyte uptake than ASOs. It is imperative to understand how much these variables contribute to drug clearance and how they are affected by various diseases [114,183]. We also hope that research on the delivery of GalNAc-siRNA drugs by oral instead of subcutaneous administration can be explicitly achieved when treating patients with chronic disease. In summary, although this technology still faces several challenges, its rapid emergence and success in a wide range of liver-based conditions have absolutely changed the standpoint for siRNA therapies targeting hepatocytes.

## Figures and Tables

**Table 1:** Reported approved/Phase III clinical trials of siRNA-based drugs.

Drug Name	Delivery Platform/Targeting Ligand	Disease/Targeting Gene	Company	Updated Status
Onpattro (Patisiran)	LNP-siRNA	TTR-mediated amyloidosis (Transthyretin)	Alnylam	FDA. approval (10/10/2018)
Givlaari (Givosiran)	GalNAc-siRNA	Acute hepatic porphyria (delta-aminolevulinate synthase 1)	Alnylam	FDA approval (11/20/2019)
Oxlumo (Lumasiran)	GalNAc-siRNA	Primary hyperoxaluria type 1 (hydroxy acid oxidase 1)	Alnylam	FDA approval (11/23/2020)
Leqvio (Inclisiran)	GalNAc-siRNA	Hypercholesterolemia (proprotein convertase subtilisin/Kexin type 9)	Alnylam Novartis	FDA approval (12/22/2021)
Vutrisiran (ALN-TTRSC02)	GalNAc-siRNA	TTR-mediated amyloidosis (Transthyretin)	Alnylam	Phase III
Fitusiran (ALN-AT3SC)	GalNAc-siRNA	Haemophilia A and B and rare blood disorders (antithrombin)	Alnylam, Sanofi, Genzyme	Phase III
Nedosiran (DCR-PHXC)	GalNAc-siRNA	Primary hyperoxaluria (lactate dehydrogenase A)	Alnylam, Dicerna	Phase III
Teprasiran (QPI-1002)	None	Acute kidney injury (tumor protein)	Quark, Novartis	Phase III
Cosdosiran (QPI-1007)	None	NAION and glaucoma (Caspase 2)	Quark,	Phase III
Tivanisiran (SYL1001)	None	Ocular pain and dry eye disease (TRPV1)	Sylentis	Phase III

**Table 2:** Chemical structures and names of ionizable lipids.

Chemical Structure of Ionizable Lipids	Name		Reference
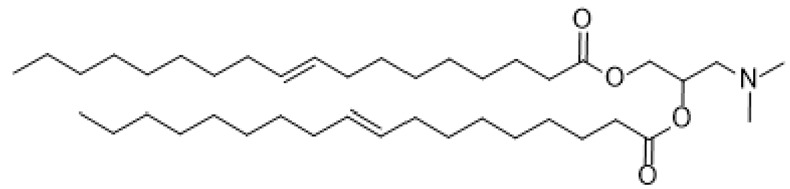	DODAP	1,2-dioleoyl-3-dimethylammonium propane	[53]
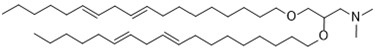	DLinDMA	1,2-dilinoleyloxy-*N*,*N*-dimethyl-3-aminopropane	[52,56]
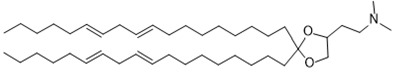	DLin-KC2-DMA	2,2-dilinoleyl-4-dimethylaminoethyl-[1,3]-dioxolane	[57]
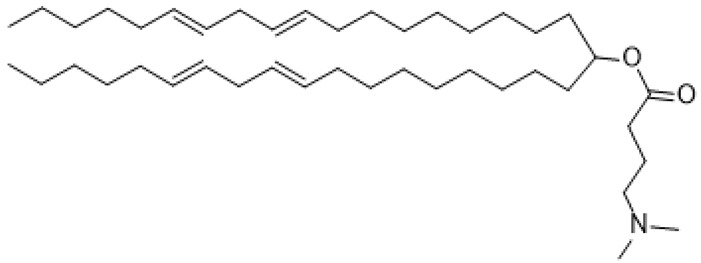	DLin-MC3-DMA	(6*Z*,9*Z*,28*Z*,31*Z*)-heptatriaconta-6,9,28,31-tetraen-19-yl 4-(dimethylamino) butanoate	[51,58,59]

**Table 3:** The current registered clinical studies on inclisiran [169] (* recruiting (green color), ** active, not recruiting (red color), *** completed (blue color), **** not-recruiting (brown color), ***** enrolling by invitation (purple color)).

NCT Number/Other IDs	Study Title	Status/Study Type/Phases	Study Design	Start/ Completion Date	NCT Number/Other IDs	Study Title	Status/Study Type/Phases	Study Design	Start/ Completion Date
NCT04929249/ CKJX839A1US02	Randomized study to evaluate the effect of an “inclisiran first” implementation strategy compared to usual care in patients with atherosclerotic cardiovascular disease and elevated LDL-C despite receiving maximally tolerated Statin Therapy (VICTORION-INITIATE)	* Recruiting/Interventional/Phase 3	None (Open Label), Primary Purpose: Treatment	25-Jun-2021 to 30-Jun-2023	NCT03814187/ MDCO-PCS-17-05/CKJX839A12306B/2017-003092-55	Trial to assess the effect of long-term dosing of inclisiran in subjects with High CV risk and elevated LDL-C (ORION 8)	** Active, not recruiting/Interventional/Phase 3	None (Open Label), Primary Purpose: Treatment	16-Apr-2019 to 31-Dec-2023
NCT05118230/ CKJX839A1CN01	Study to assess the real-world effectiveness of inclisiran in Chinese adult patients with primary hypercholesterolemia or mixed dyslipidemia	* Recruiting/Observational/NA	Observational Model: Cohort, Time Perspective: Prospective	9-Dec-2021 to 30-Sep-2024	NCT05362903/ CKJX839A1DE01	A non-interventional implementation study to evaluate treatment with inclisiran (Leqvio) and other lipid lowering treatments in a real-world setting	* Recruiting/Observational/NA	Observational Model: Cohort, Time Perspective: Prospective	28-Jan-2023 to 31-Jan-2025
NCT03159416/ MDCO-PCS-16-03	A study of inclisiran in participants with renal impairment compared to participants with normal renal function (ORION-7)	*** Completed/Interventional/Phase 1	None (Open Label), Primary	22-Jun-2017 to 24-Mar-2018	NCT04873934/ CKJX839A1US01	Management of LDL-cholesterol with inclisiran + usual care compared to usual care alone in participants with a recent acute coronary syndrome	* Recruiting/Interventional/Phase 3	Allocation: Randomized, Intervention Model: Parallel Assignment, Masking:	24-Jun-2021 to 20-Mar-2024
NCT03060577/ MDCO-PCS-16-01	An extension trial of inclisiran compared to evolocumab in participants with cardiovascular disease and high cholesterol (ORION-3)	*** Completed/Interventional/Phase 2	Allocation: Non-Randomized, Intervention Model: Parallel Assignment, Masking: None (Open Label), Primary Purpose: Treatment	24-Mar-2017 to 17-Dec-2021	NCT05399992/ CKJX839A12401	Study evaluating effectiveness and adherence of inclisiran plus standard of care (SoC) lipid-lowering therapy compared to SoC in ASCVD	**** Not-recruiting/Observational/NA	Observational Model: Cohort, Time Perspective: Prospective	30-Aug-2022 to 1-Apr-2022
NCT04774003/ CKJX839A12105	Study of pharmacokinetics, pharmacodynamics, safety, and tolerability of inclisiran in Chinese participants with elevated serum LDL-C (ORION-14)	*** Completed/Interventional/Phase 1	Allocation: Randomized, Intervention Model: Parallel Assignment, Masking: Quadruple (Participant, Care Provider, Investigator, Outcomes Assessor), Primary Purpose: Treatment	26-Feb-2021 to 18-Oct-2021	NCT04659863/ CKJX839C12302/2020-002755-38	Study to evaluate efficacy and safety of inclisiran in adolescents with homozygous familial hypercholesterolemia (ORION-13)	* Recruiting/Interventional/Phase 3	Allocation: Randomize, Intervention Model: Parallel Assignment, Masking: Double (Participant, Investigator), Primary Purpose: Treatment	15-Feb-2021 to 9-Dec-2024
NCT04652726/ CKJX839C12301/2020-002757-18	Study to evaluate efficacy and safety of inclisiran in adolescents with heterozygous familial hypercholesterolemia (ORION-16)	* Recruiting/Interventional/Phase 3	Allocation: Randomized, Intervention Model: Parallel Assignment, Masking: Double (Participant, Investigator), Primary Purpose: Treatment	27-Jan-2021 to 9-Dec-2024	NCT04807400/ CKJX839A1GB01/2020-004401-31	Study in primary care evaluating inclisiran delivery implementation + enhanced support	** Active, not recruiting/Interventional/Phase 3	Allocation: Randomized, Intervention Model: Parallel Assignment, Masking: None (Open Label), Primary Purpose: Health Services Research	7-Jul-2021 to 30-Jan-2023
NCT05192941/ CKJX839A12402/2021-003759-40	Study of efficacy, safety, tolerability, and quality of life of inclisiran (kjx839) vs. placebo, on top of on-going individually optimized lipid-lowering therapy, in participants with hypercholesterolemia	* Recruiting/Interventional/Phase 4	Allocation: Randomized, Intervention Model: Parallel Assignment, Masking: Quadruple (Participant, Care Provider, Investigator, Outcomes Assessor), Primary Purpose: Treatment	8-Apr-2022 to 4-Feb-2025	NCT04666298/ CKJX839A11201	Study of efficacy and safety of inclisiran in Japanese participants with high cardiovascular risk and elevated LDL-C	** Active, not recruiting/Interventional/Phase 1	Allocation: Randomized, Intervention Model: Parallel Assignment, Masking: Quadruple (Participant, Care Provider, Investigator, Outcomes Assessor), Primary Purpose: Treatment	29-Jan-2021 to 16-Oct-2021
NCT03851705/ MDCO-PCS-17-02/CKJX839A12302	A study of inclisiran in participants with homozygous familial hypercholesterolemia (HoFH) (ORION-5)	*** Completed/Interventional/Phase 3	Allocation: Randomized, Intervention Model: Parallel Assignment, Masking: Double (Participant, Investigator), Primary Purpose: Treatment	6-Feb-2019 to 9-Sep-2021	NCT03399370/ MDCO-PCS-17-04	Inclisiran for participants with atherosclerotic cardiovascular disease and elevated low-density lipoprotein Cholesterol (ORION-10)	*** Completed/Interventional/Phase 3	Allocation: Randomized, Intervention Model: Parallel Assignment, Masking: Double (Participant, Care Provider), Primary Purpose: Treatment	21-Dec-2017 to 17-Sep-2019
NCT03400800/ MDCO-PCS-17-08	Inclisiran for subjects with ASCVD or ASCVD-risk equivalents and elevated low-density lipoprotein cholesterol	*** Completed/Interventional/Phase 3	Allocation: Randomized, Intervention Model: Parallel Assignment, Masking: Double (Participant, Care Provider), Primary	1-Nov-2017 to 27-Aug-2019	NCT03397121/ MDCO-PCS-17-03/2017-002472-30	Trial to evaluate the effect of inclisiran treatment on low density lipoprotein cholesterol (LDL-C) in subjects with heterozygous familial hypercholesterolemia (HeFH) (ORION-9)	*** Completed/Interventional/Phase 3	Allocation: Randomized, Intervention Model: Parallel Assignment, Masking: Double (Participant, Care Provider), Primary Purpose: Treatment	28-Nov-2017 to 17-Sep-2019
NCT04765657/ CKJX839A12307	Study of efficacy and safety of inclisiran in asian participants with atherosclerotic cardiovascular disease (ASCVD) or ASCVD high risk and elevated low density lipoprotein cholesterol (LDL-C)	** Active, not recruiting/Interventional/Phase 3	Allocation: Randomized, Intervention Model: Parallel Assignment, Masking: Quadruple (Participant, Care Provider, Investigator, Outcomes Assessor), Primary Purpose: Treatment	1-Mar-2021 to 30-Sep-2022	NCT03705234/ CTSU_MDCO_PCS-17-01	A randomized trial assessing the effects of inclisiran on clinical outcomes among people with cardiovascular disease (ORION-4)	* Recruiting/Interventional/Phase 3	Allocation: Randomized, Intervention Model: Parallel Assignment, Masking: Quadruple (Participant, Care Provider, Investigator, Outcomes Assessor), Primary Purpose: Prevention	30-Oct-2018 to Dec 2049
NCT05360446/ CKJX839D12303, 2021-004601-47	Coronary computed tomography study to assess the effect of inclisiran in addition to maximally tolerated statin therapy on atherosclerotic plaque progression in participants with a diagnosis of non-obstructive coronary artery disease	* Recruiting/Interventional/Phase 3	Allocation: Randomized, Intervention Model: Parallel Assignment, Masking: Triple (Participant, Care Provider, Investigator), Primary Purpose: Treatment	8-Jul-2022 to 5-Jun-2025	NCT05004675/ LIB003-012	Trial to evaluate efficacy and safety of lib003 and inclisiran in high-risk CVD Patients	***** Enrolling by invitation/Interventional/Phase 3	Allocation: Randomized, Intervention Model: Parallel Assignment, Masking: Quadruple (Participant, Care Provider, Investigator, Outcomes Assessor), Primary Purpose: Treatment	20-Jun-2022 to31-0ct-2023
NCT05030428/ CKJX839B12302	Study of inclisiran to prevent cardiovascular (CV) events in participants with established cardiovascular disease	* Recruiting/Interventional/Phase 3	Allocation: Randomized, Intervention Model: Parallel Assignment, Masking: Double (Participant, Investigator), Primary Purpose: Treatment	23-Nov-2021 to 13-Oct-2027	NCT02314442/ ALN-PCSSC-001	A phase 1 study of an investigational drug, aln-pcssc, in subjects with elevated low density lipoprotein cholesterol (LDL-C)	*** Completed/Interventional/Phase 1	Allocation: Randomized, Intervention Model: Parallel Assignment, Masking: Single (Participant), Primary Purpose: Treatment	Dec-2014 to Nov-2015
NCT02597127/ MDCO-PCS-15-01	Trial to evaluate the effect of ALN-PCSSC treatment on low density lipoprotein cholesterol (LDL-C) (ORION-1)	*** Completed/Interventional/Phase 2	Allocation: Randomized, Intervention Model: Parallel Assignment, Masking: Double (Participant, Investigator), Primary Purpose: Treatment	Jan-2016 to 7-Jun-2017	NCT05438069/ 2021-2429	German inclisiran network: retrospective registry of patients being treated with the siRNA inclisiran in Germany	* Recruiting/Observational	Observational Model: Cohort, Time Perspective: Retrospective	11-Dec-2020 to 11-Dec-2025
NCT01437059/ ALN-PCS02-001	Trial to evaluate safety and tolerability of ALN-PCS02 in subjects with elevated LDL-Cholesterol (LDL-C) (ORION 2)	*** Completed/Interventional/Phase 1	Allocation: Randomized, Intervention Model: Parallel Assignment, Masking: Single (Participant), Primary Purpose: Treatment	Sep 2011-Sep 2012	NCT02963311/ MDCO-PCS-16-02	A study of ALN-PCSSC in participants with homozygous familial hypercholesterolemia (HoFH)	*** Completed/Interventional/Phase 2	Allocation: N/A, Intervention Model: Single Group Assignment, Masking: None (Open Label), Primary Purpose: Treatment	13-Dec to 8-Oct-2018

* low density lipoprotein cholesterol-LDL-C; homozygous familial hypercholesterolemia-HoFH; cardiovascular disease-CVD; atherosclerotic cardiovascular disease-ASCVD.

## Data Availability

Not applicable.
